# Clinical trials with MRNA electroporated dendritic cells for stage III/IV melanoma patients

**DOI:** 10.1186/2051-1426-3-S2-P211

**Published:** 2015-11-04

**Authors:** Sofie Wilgenhof, Jurgen Corthals, Carlo Heirman, Bart Neyns, Kris Thielemans

**Affiliations:** 1 Vrije Universiteit Brussel, Brussels, Belgium; 2 Universitair Ziekenhuis Brussel, Brussels, Belgium; 3 Vrije Universiteit Brussels, Brussels, Belgium

## Background

TriMixDC-MEL consists of autologous monocyte-derived DC that are electroporated with synthetic mRNA encoding CD40 ligand, a constitutively active TLR4, CD70 and fusion proteins of DC.LAMP with 4 melanoma associated antigens (MAGE-A3, MAGE-C2, tyrosinase and gp100).

## Methods

TriMixDC-MEL was investigated in patients (pts) with pretreated advanced melanoma, either as a single agent (Phase Ib; NCT01066390) or combined with IPI (Phase II; NCT01302496; 10 mg/kg q3wks x4) and also in melanoma pts who are disease free following local treatment of macrometastases. TriMixDC-MEL was administered by the IV and ID-route (4 to 5 admin; 4.10^6^-ID/20.10^6^-IV).

## Results

In pts with unresectable AJCC stage III or IV melanoma, respectively 15 and 39 pts were treated in the Phase Ib and the –II trial. DC-related AEs consisted of local inflammatory skin reactions at the DC-injection site (all pts), grade (gr) 2 acute post-IV injection chills in 20% and 38%, and gr 1-2 flu-like syndrome in 53% and 85% of pts treated respectively with DC or DC+IPI. Grade 3 or 4 irAEs occurred in 36% of DC+IPI treated pts. ORR for DC: 27% (2 CR, 2 PR; 3 are ongoing after > +51 mths) and 38% for DC+IPI (8 CR, 7 PR; 8 are ongoing after > +16, mths). Median PFS and OS are respectively 5 (95% CI 0–10) and 14 mths (95% CI 5–23) for DC and 6.2 (95% CI 2-10) and 13 mths (95% CI, 9-18) for DC+IPI. The 1, 2 and 3y OS% for DC+IPI were: 59% (95% CI 43-74), 38% (95% CI, 23-53), and 34% (95% CI, 19-50). Treatment with TriMixDC-MEL, especially in combination with IPI, is tolerable and results in a high rate of durable tumor responses. In the adjuvant setting (NCT01676779), 41 patients were randomized between the TriMixDC-MEL treatment arm (n=21) and control-arm (n=20). Baseline characteristics were well balanced between both groups. After a median follow-up of 18 mths (range 5 to 30 mths) 20 patients experienced a non-salvageable melanoma recurrence (6 on the DC- and 14 on the control-arm). The rate of patients who were disease-free at 1 year (evaluable population = 35 patients) was higher in the TriMixDC-MEL treated group (65% [95%CI 42-87] vs. 34% [13-55]). TriMixDC-MEL was well tolerated (no grade >3 AE).

## Conclusions

The results of this non-comparative randomized controlled Phase II clinical trial of TriMixDC-MEL ID/IV versus observation support the further evaluation of TriMixDC-MEL as a well-tolerated adjuvant therapy for melanoma patients following the resection of macrometastases.

